# Promoting inclusion, diversity, and equity in pain science

**DOI:** 10.1080/24740527.2022.2161272

**Published:** 2023-02-22

**Authors:** Tonya M. Palermo, Karen Deborah Davis, Didier Bouhassira, Robert W. Hurley, Joel D. Katz, Francis J. Keefe, Michael Schatman, Dennis C. Turk, David Yarnitsky

**Affiliations:** Department of Anesthesiology & Pain Medicine, University of Washington, Seattle, Washington, USA; Department of Surgery and Institute of Medical Science, Krembil Brain Institute, University Health Network; University of Toronto, Toronto, Ontario, Canada; Inserm U987, APHP, UVSQ, Paris-Saclay University, Ambroise Pare Hospital, Boulogne-Billancourt, France; Department of Anesthesiology, Neurobiology and Anatomy, Wake Forest University School of Medicine, Winston–Salem, North Carolina, USA; Department of Anesthesiology & Pain Medicine, University of Washington, Seattle, Washington, USADepartment of Neurology, Rambam Medical Center, and Laboratory of Clinical Neurophysiology, Technion Faculty of Medicine, Haifa, Israel; Department of Psychiatry and Behavioral Sciences, Duke University School of Medicine, Durham, North Carolina, USA; Department of Anesthesiology, Perioperative Care, and Pain Medicine, NYU Grossman School of Medicine, New York, New York, USA; Department of Population Health–Division of Medical Ethics, NYU Grossman School of Medicine, New York, New York, USA; Department of Neurology, Rambam Medical Center, and Laboratory of Clinical Neurophysiology, Technion Faculty of Medicine, Haifa, Israel

Science and medicine have a long and troubling history of reinforcing racist, antiethnic, and sexist attitudes and beliefs, as well as ignoring and mistreating marginalized people. In their editorial in *Nature*,^1^ titled “Science Must Overcome Its Racist Legacy,” Nobles and colleagues described how the scientific enterprise has reinforced racist beliefs and cultures over time. For example, the authors discussed the impact of colonization on racism, including how apartheid, imperialism, colonialism, slavery, and eugenics were memorably endorsed by science. They then asserted that acknowledging and learning this history is necessary if we are to restore, rebuild, and avoid perpetuating these injustices. Similarly, there is a long history of exclusion of women from science and medicine as a profession due to structural discrimination as well as exclusion of women from research participation in clinical trials to develop pharmacologically based therapeutics.^2^

The science of pain is no exception. As Bourke^3^ explored in an article on the history of pain sensitivity, misconceptions about pain sensitivity put forth by physicians and scientists from 1800 to 1965 reified existing social hierarchies. It was believed that sensitivity to painful stimuli was linked to different personalities that were biologically defined by race, sex, or religion, thereby reinforcing existing racist and sexist science. Unfortunately, myths regarding pain and implicit biases persist, with some studies suggesting that gender stereotypes influence pain estimates due to beliefs that women are more emotive and men are more stoic.^4^ This can contribute to an underestimation of pain in female persons, a finding reinforced by studies on estimating pain in both adults and children.^5^

Racial misconceptions regarding heightened pain tolerance among Blacks also continue to persist. For example, a recent study determined that White medical students and laypeople endorsed beliefs that there were biological differences (i.e., having thicker skin) that accounted for higher pain tolerance in Black individuals, and these beliefs were linked with racial bias in pain treatment recommendations.^6^ Misconceptions and biases pertaining to age and development have also influenced pain treatment and pain science. For example, until the 1980s, an erroneous belief that infants did not feel pain resulted in major surgeries being performed on infants without anesthesia.^7^ At the other end of the age spectrum, there has been an exclusion of adults over age 65 years from studies evaluating pain therapies.^8^

Indeed, disparities in pain management and quality of care are profound. The undertreatment of pain in racial minority groups was brought to the forefront 3 decades ago^9^; nevertheless, pain care continues to be strongly influenced by biases and misconceptions about pain. An extensive body of research documents pain inequities by sociodemographic characteristics (e.g., racialized group, ethnicity, age, sex, gender identity, socioeconomic position, regional location)^10^ and by sexual minority status.^11^ Tragically, in many instances, the individuals who experience the greatest burden from pain are the same individuals who are underrepresented in studies that seek to understand and alleviate pain.^10^ Thus, recent calls to action have been put forth to make pain research more inclusive and to use anti-racist principles in the conduct, reporting, and interpretation of pain research.^12–14^

As editors of journals in the field of pain research and medicine (*Canadian Journal of Pain, Clinical Journal of Pain, European Journal of Pain, Journal of Pain, Journal of Pain Research, PAIN, Pain Medicine*, and *PAIN Reports*), we are united in our commitment to eliminate disparities in pain science and to provide an inclusive environment for scholarship and publishing. Therefore, we endorse calls for greater inclusion in the conduct of science and also in scholarship regarding the reporting, review, and dissemination of evidence.^15^ Reviewers and editors serve an important gate-keeping role and can help bring attention to racist and sexist beliefs and promote fairness and equity, while helping to facilitate trust and restore power imbalances. We acknowledge that concerted attention is needed to increase representation in pain research to support inclusion, diversity, and equity in the articles published in pain journals. We also acknowledge that the ideas in this editorial reflect decades of work by health equity scholars and we recognize and honor their contributions to the field (please see the reference list, which, though not exhaustive, highlights work from a number of these scholars).

Below we describe and endorse four interrelated principles to guide authors in their article submissions to our journals. These principles can also inform journal policies and serve as a guide for reviewers, editors, and publishers.

## General Principles

### Promote Inclusive and Representative Scholarship and Fair, Unbiased Reviews

#### For Authors

There is strong evidence that women and people of color are undercited in the scientific literature across a large range of topic areas.^16^ This lack of acknowledgment, recognition, and valorization of ideas contributes to inequities in pain science. We ask authors to think carefully about the diversity of their citations of the literature and whether they have consciously or unconsciously omitted work by authors based on their sex, gender, race, ethnicity, geographic location, or reputation of institution where research was conducted. At this time, we are aware of one tool developed to understand representativeness of citations, specifically gender citation balance, the Gender Citation Balance Index tool (“GCBI-alizer”; https://postlab.psych.wisc.edu/gcbialyzer/) that was introduced in 2021 in the *Journal of Cognitive Neuroscience*.^17,16^ We encourage the development of additional software in the future to assist authors in recognizing the diversity of their citations.

For reviewers, editors, and publishers: We encourage, embrace, and support diversity among our reviewers, editors, and editorial boards to provide a broader perspective of ideas and promote high-quality and more generalizable science. We aim to nurture the growth of a diverse pipeline of scientists who are prepared for future editorial leadership.

We ask reviewers to think carefully about biases and assumptions that they may hold (e.g., knowing the author’s gender, country of origin, reputation of their institution) that affect their evaluation of manuscripts, including their opinions on the accuracy and reliability of findings. We encourage the use of available tools to help reviewers understand and challenge common biases and assumptions.^18^

We applaud the work of The Joint Commitment for Action on Inclusion and Diversity in Publishing^19^ led by the Royal Society of Chemistry, which brings together 52 publishers representing more than half (over 15,000 journals) of the world’s peer-reviewed academic journals. They are collaborating to collect diversity data from authors, editors, and reviewers about their race, ethnicity, and gender. Some journals have already begun to implement this data collection, and soon these data on the diversity of journals on these dimensions will be available globally. This is a critical starting point that can be used to create benchmarks to identify where change is needed and to measure outcomes of actions taken by journals to increase diversity and inclusion. We recognize that additional data and metrics are needed to help to understand other areas of diversity (e.g., by geographic location, socioeconomic status, disability, age).

### Use Language that Is Inclusive and Minimizes Bias

#### For Authors

As suggested in the American Psychological Association general principles^20,^ for reducing bias, “Choose labels with sensitivity, ensuring that the individuality and humanity of people are respected; https://apastyle.apa.org/style-grammar-guidelines/bias-free-language/general-principles.” The International Committee of Medical Journal Editors (ICMJE)^21^ recommends that authors use “neutral, precise, and respectful language to describe study participants and avoid the use of terminology that might stigmatize participants (p. 16).” Given the international scope of our journals, we recognize that terminology can be particularly challenging given differing meanings of terms globally and limitations of direct translation. In general, we suggest use of language that is as inclusive and bias-free as possible. There are available style guides (e.g., the APA style and grammar guidelines for bias-free language^20^ and the *AMA Style Manual*: inclusive language^22^) that should be consulted for best practices. These style guides cover a variety of issues (describing sex and gender, personal pronouns, race and ethnicity, socioeconomic status, sexual orientation, and terms for describing people with diseases, disorders, or disabilities, as well as inclusive practices for presenting demographic data) and provide both problematic and preferred examples of language to convey respect and inclusivity.

Recommendations for describing people with painful conditions would include person-first terms such as “people living with chronic pain” or “patients with chronic pain” instead of the term “chronic pain patients.” Inclusive language should avoid negative and condescending terminology. Another aspect of inclusive language for authors to follow is to avoid using terms for a particular group (e.g., Whites, men, able-bodied) that suggest they are the “normal” comparator and that other groups (e.g., racialized, women, disabled) are the “abnormal” comparator or deviant. Rather, authors should use terms that maintain equality. We encourage all authors to become familiar with these guides and to also be aware that they are periodically updated as societal norms continue to evolve.

#### For Reviewers and Editors

We encourage reviewers and editors to also be familiar with inclusive and bias-free language and to work toward identifying instances of biased and condescending language in their reviews and editorial processes. Further, we encourage providing authors with constructive suggestions specifying how to make their language more inclusive. Editors can familiarize themselves with these guidelines in order to set future policies for their journals and to ensure that communications coming from their journals follow these same principles.

### Include Representative Populations in Pain Research and Comprehensively Report Data for Demographic Variables

#### For Authors

Inclusion is important across all types of pain research. It is well documented that women (and females in preclinical research) have been underrepresented (or omitted) in many areas of research in human-based studies and clinical trials, as well as animal studies. For example, in an examination of animal studies published in *PAIN* over a 10-year period, Greenspan and colleagues^23,^ found that only male animals were included for 79% of the studies. To address sex and gender bias in research, the Sex And Gender Equity in Research (SAGER) international guideline was established,^24^ which provides recommendations on not only the conduct of more inclusive science but reporting of sex and gender information in study design, data analysis, results, and interpretation of findings. Consistent with these guidelines, in animal experiments, we call for the inclusion of both male and female animals in studies unless there is a solid scientific rationale against doing so.

In human studies, inclusion of representative groups by sex, gender, age, race, and ethnicity, among other forms of diversity, is key to gaining knowledge to address pain assessment and treatment inequities. Our guidance is also consistent with the ICMJE recommendations on the conduct, reporting, editing, and publication of scholarly work in medical journals,^21^ which strongly encourages clinical researchers to aim for inclusion of representative populations in their studies and to also provide relevant demographic variables whenever possible.

We encourage reporting demographic variables in as detailed a fashion as possible to describe human participants, as well as description of sex of cells or tissue cultures and animals. The SAGER guidelines recommend careful use of terminology for sex (biological variable) and gender (socially constructed variable) to reduce confusing both terms. We recognize, however, that there will be differences in availability and suitability of certain demographic data such as race, ethnicity, sex, or gender, based on different countries. Moreover, measurement frameworks for culture, race, and ethnicity differ worldwide and thus may not be commonly or consistently presented or even valid. However, consistent with ICJME recommendations, whenever possible, authors should define how they determined sex, gender, race, or ethnicity and how data were collected (e.g., self-report, medical record). If race or ethnicity data were not collected, authors should explain why not. In studies in which representative samples are not included, we encourage authors to reflect on the limitations of their findings and approaches that may improve inclusion in future research.

We encourage authors to devise strategies to address inclusion. For example, involving key stakeholders (e.g., people with lived experience of pain, pain care providers) and rights holders (e.g., Indigenous partners) is one recommended strategy that may help address issues related to recruitment of diverse populations. For further details, see the comprehensive study planning and design checklists offered in Janevic et al.^10^ to increase inclusion in pain research.

#### For Reviewers and Editors

We encourage reviewers to consider the representativeness of study samples and to provide constructive comments on how sample representativeness and inclusion may influence interpretations of study findings. Reviewers may also help identify missing details in the reporting of demographic variables. Editors should carefully weigh these concerns in their editorial decisions, considering how interpretability of findings and generalizability affect the overall manuscript quality.

### Report Demographic Variables and Use Social Frameworks for Interpretations

#### For Authors

We call on authors to include data analyses on disaggregated data based on demographic variables to facilitate future pooling and meta-analysis and to report results separately by sex (e.g., analyze clinical trial outcome data separately for men and women). This is consistent with the Canadian Institutes of Health Research guidelines for sex- and gender-based analysis^25^ as well as the ICMJE recommendations^21^ to separate reporting of data by demographic variables, such as age and sex, unless there are compelling reasons not to do so.

We also encourage authors to carefully consider their analysis of and interpretations of data relating to differences in pain based on sex, gender, race, and ethnicity using frameworks that account for cultural and social experiences. Sex differences in pain are now clearly documented,^26,27^ and careful attention to analyzing sex differences in animal and human experiments has, helped to identify sex-specific mechanisms of acute and chronic pain processing. Some funding agencies now mandate inclusion of sex as a biological variable in preclinical research, which is associated with increasing numbers of studies investigating sex differences in pain and analgesia.^28^ However, with this increased focus comes the risk that research in this important area will be misinterpreted and misrepresented. Fallacies regarding sex differences have been described^29^; for example, that sex differences are caused only by genetic or hormonal influences rather than by social experience. Such arguments ignore the effects of sex-specific experience and can unfortunately reinforce sex stereotypes and limit understanding of sex and gender differences in pain.^30^

Misinterpretation of findings pertaining to race and ethnicity is common. Historically, authors have treated race and ethnicity as biological constructs in their analyses and in their interpretation and discussion of findings (e.g., that differences between racial groups reflect biological variation rather than inequities based on social or cultural factors). However, there is compelling and convincing evidence that is now becoming commonly accepted that race and ethnicity are not biological categories but rather are socially defined.^31^ Unfortunately, the consequences of longstanding misinterpretations have been severe and have excluded individuals from health care and research resources (e.g., assuming that any differences in health outcomes between Black people and White people are based on fundamental differences in biology and that health care should be provided differently).^32^

Consistent with guidance published by Flanagin et al.^33^ for the JAMA Network, we encourage authors to consider and include measures of cultural and social experiences, such as discrimination, poverty, and access to care, as well as the intersectionality of race, sex, and ethnicity with these other factors. Authors are encouraged to not report race or ethnicity in isolation but to include a combination of these other sociodemographic and social determinants to further advance understanding of racial, sex, and ethnic disparities in pain.

#### For Reviewers and Editors

We encourage reviewers to consider how data are presented and whether current norms allow for subgroup analyses by demographic variables. Reviewers should consider how analyses of racial, ethnic, gender, or sex differences are presented and interpreted. Reviewers and editors should be aware of biases that reinforce racist, ethnic, and sexist stereotypes. When examining data on racial, ethnic, gender, and sex differences in pain, consideration of whether appropriate analyses have been presented to allow understanding of variation and overlap between groups (as opposed to a difference only) and discussion of the explanatory factors that covary with sex, gender, race, or ethnicity strengthen the meaning and generalizability of findings. Reviewers can encourage authors to consider cultural and social experiences in interpretations of findings if these have not been explored to improve the quality of research. We ask that editors carefully weigh these concerns when making decisions about the appropriateness of publication of a manuscript.

Each of the represented pain journals are developing policies to implement these four principles using its own strategies. We encourage authors to consult the journals’ author guidelines and learn more about the actions of each journal over the coming year. We recognize that some authors and peer reviewers will find our recommendations onerous and will question their value. These principles are not simply a matter of avoiding offending marginalized groups but are fundamental to improving the quality of pain science to offer greater precision, transparency, and equity in order to develop treatments that improve the lives of all people experiencing pain. There is a tremendous opportunity to affect change at this time of heightened knowledge of how to eliminate disparities in pain science, which our research predecessors in the context of their surrounding culture did not have an opportunity to do.

In conclusion, we are committed to the promotion of inclusion, diversity, and equity in pain science. We believe that harmonizing principles of inclusion, equity, diversity, and anti-racism across our journals will yield greater validity of research and thereby have a larger impact on pain science than will individual efforts in isolation. These steps are admittedly long overdue, and we hope that the entire pain scientific community will support these principles in their roles as investigators, authors, reviewers, and editors.
